# Liquid‐Phase Syngas‐To‐Methanol at Low Temperature: Mixed Alcohol Solvent‐Controlled Pathways for Circular Carbon Manufacturing

**DOI:** 10.1002/cssc.202502414

**Published:** 2026-03-03

**Authors:** Guanfu Liu, Helena Hagelin‐Weaver, Pratap Pullammanappallil, Ziynet Boz, Ana Martin‐Ryals, Bruce Ari Welt

**Affiliations:** ^1^ Agricultural & Biological Engineering Department Packaging Engineering Program University of Florida/IFAS Gainesville Florida USA; ^2^ Chemical Engineering Department University of Florida Gainesville Florida USA

## Abstract

Methanol is a primary platform chemical for circular manufacturing. Low‐temperature liquid‐phase methanol synthesis provides an energy‐efficient route for valorizing syngas under mild conditions. In this study, we explore the effect of mixed alcohol solvents—specifically 2‐butanol and isopropanol—on methanol production over a commercial CuO/ZnO/Al_2_O_3_ catalyst at 170°C and 5 MPa. These two alcohols were selected based on prior reports of high reactivity, with 2‐butanol showing superior performance but at a higher cost. A series of reactions with varying 2‐butanol content (0%–100%) revealed a nearly linear relationship between solvent composition and methanol yield, suggesting minimal interaction between the two alcohols. Product analysis identified isopropyl formate as the only observable ester intermediate when isopropanol was present, while no 2‐butyl formate was detected. These findings clarify how solvent choice influences intermediate stability and methanol productivity, providing guidance for process optimization. Beyond reaction chemistry, this approach integrates with Regenerative Robust Gasification (RRG), which converts heterogeneous organic waste streams into syngas upstream and channels methanol downstream into products, polymers, packaging, and other circular products, advancing circular economy strategies under industrially relevant conditions.

## Graphical abstract

Low‐temperature liquid‐phase methanol synthesis from syngas was demonstrated over CuO/ZnO/Al_2_O_3_ catalysts using mixed alcohol solvents. Solvent composition controls ester intermediate and methanol productivity.

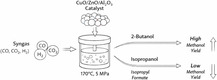

## 1 Introduction

Methanol (MeOH) is a primary C1 platform chemical that underpins multiple value chains in chemicals and materials, including olefin production via methanol‐to‐olefins (MTO) routes, as well as oxygenates such as acetic acid and dimethyl ether; this breadth of downstream pathways makes MeOH an effective molecular hub for circular manufacturing [1, 2, 3, 4, 5, 6, 7].

Within petrochemical infrastructures, MTO technologies are commercially deployed and increasingly optimized, providing a mature route from MeOH to polyethylene‐ and polypropylene‐based packaging resins relevant to consumer packed goods (CPG) supply chains [8, 9].

Regenerative Robust Gasification (RRG) provides the systems context for integrating waste streams with MeOH‐centered value chains. RRG uses high‐temperature, feedstock‐agnostic conversion of heterogeneous wastes (including mixed plastics and complex packaging) to produce specification syngas, decoupling feedstock variability from back‐end synthesis [10]. Technoeconomic and design studies corroborate the feasibility of converting plastics and municipal solid waste (MSW) to methanol (MeOH) via gasification, syngas clean‐up, and synthesis [11, 12]. Placing MeOH synthesis within RRG thus links upstream waste heterogeneity to downstream product diversity through a single, fungible intermediate.

Conventional MeOH synthesis employs Cu/ZnO/Al_2_O_3_ catalysts at 220°C–300°C and 5–10 MPa, where equilibrium and heat removal limit single‐pass productivity [13, 14]. By contrast, low‐temperature liquid‐phase methanol synthesis (LT‐LPMS) in alcoholic solvents (≤200°C) leverages solvent participation via formate‐type intermediates that hydrogenate to MeOH under mild conditions, enabling process intensification without catalyst redesign [15, 16, 17, 18, 19, 20, 21]. Solvent identity governs intermediate stability and hydrogenolysis kinetics; secondary alcohols such as 2‐butanol often exhibit superior apparent productivity compared to other solvents under similar conditions [22, 23, 24, 25, 26, 27, 28].

For energy applications within RRG, it is essential to distinguish between biogenic and fossil carbon fractions. Under the U.S. Renewable Fuel Standard (RFS), only the biogenic portion of MSW‐derived fuels qualifies as renewable; producers typically verify biogenic content using ASTM D6866 radiocarbon analysis and operate under EPA‐approved waste separation plans [29, 30]. Methodologies for allocating MSW energy to biogenic versus nonbiogenic shares are documented by the Energy Information Administration [31]. Similarly, California's Low Carbon Fuel Standard (LCFS) requires auditable attribution and carbon‐intensity reporting for waste‐derived fuels [32]. Consequently, in RRG, biogenic MeOH can be directed to fuels/power, whereas fossil‐derived MeOH is preferentially retained in circular products.

When both fossil and biogenic carbon streams are committed to long‐lived products and facilities are powered with carbon‐free energy, the infrastructure functions as an operational carbon store, embedding carbon in in‐use stocks while displacing virgin petrochemical inputs—thus complementing engineered CCUS pathways [8, 33].

Within this system's frame, the present work examines LT‐LPMS over commercial Cu/ZnO/Al_2_O_3_ at 170°C and 5 MPa using mixed alcohol solvents (2‐butanol and isopropanol). We quantify how solvent composition controls intermediate stability and MeOH yield, identify isopropyl formate as the only observable ester in isopropanol‐containing systems, and reveal a nearly linear yield trend with 2‐butanol fraction—insights that inform reaction–separation design for deploying LT‐LPMS as a modular unit in RRG [15, 19, 22].

## 2 Experimental

### 2.1 Catalyst Preparation

A commercial CuO/ZnO/Al_2_O_3_ catalyst was used throughout this study. The catalyst (DZC‐98‐5) was purchased from Shandong Dengzhuo Chemical Co., with a reported composition of CuO > 52 wt%, ZnO > 21 wt%, and Al_2_O_3_ > 8 wt%. The catalyst was supplied in pellet form and was manually ground into fine powder using a mortar prior to each experiment to ensure uniform dispersion in the liquid‐phase reaction medium.

### 2.2 Catalyst Characterization

The physicochemical properties of the commercial CuO/ZnO/Al_2_O_3_ catalyst were investigated using a series of complementary characterization techniques, including hydrogen temperature‐programmed reduction (H_2_‐TPR), X‐ray diffraction (XRD), CO pulse chemisorption, and scanning transmission electron microscopy (S/TEM).

**H_2_‐TPR** was carried out to investigate the reducibility of copper species in the commercial CuO/ZnO/Al_2_O_3_ catalyst. Experiments were performed using a ChemBET3000 instrument (Quantachrome, Inc.) equipped with a thermal conductivity detector (TCD). Approximately 50 mg of the catalyst powder was loaded into a U‐shaped quartz tube and supported on a quartz wool plug. Prior to reduction, the sample was purged with ultrahigh‐purity (UHP) argon (Airgas, Inc.) at room temperature to remove residual gases and moisture. The reduction was conducted under a flowing gas mixture of 5 vol% H_2_ in Ar at a total flow rate of 67 standard cm^3^/min (sccm). After baseline stabilization, the sample was heated from room temperature to 500°C at a rate of 10°C/min and held at 500°C for 20 min. The TPR profiles were recorded as a function of time, and the hydrogen consumption peaks were analyzed to determine the appropriate reduction temperature range for subsequent catalyst activation.

**XRD** was used to analyze the crystalline structure and phase composition of the commercial CuO/ZnO/Al_2_O_3_ catalyst. Measurements were conducted using a PANalytical X’Pert Pro diffractometer with Cu K*α* radiation (*λ* = 1.5406 Å). Catalyst powder was evenly spread onto a standard sample holder, and diffraction patterns were collected over a 2*θ* range of 20°–100° with a step size of 0.016°. The instrument was operated at 45 kV and 40 mA. The XRD patterns were also used for estimating the average crystallite size of the copper oxide and metallic copper species using the Scherrer equation:

(1) $$D=\frac{K\lambda }{\beta \cos\theta }$$

where *D* is the crystallite size, *K* is the shape factor (typically 0.9), *λ* is the X‐ray wavelength (1.5406 Å), *β* is the full width at half maximum (FWHM) in radians, and *θ* is the Bragg angle.

**CO pulse chemisorption** measurements were performed after in situ hydrogen reduction at 220°C for 1 h under a flow of 5 vol% H_2_ in Ar (67 sccm) to quantify the exposed copper surface sites. This temperature was selected based on the initial TPR data and the reduction conditions used before the reaction. Measurements were conducted on a ChemBET3000 instrument (Quantachrome, Inc.) using the same setup and quartz U‐tube as for the H_2_‐TPR experiments. Approximately 50 mg of catalyst powder was also used for these measurements. After reduction treatment at 220°C, the sample was purged with UHP helium and cooled to room temperature. CO pulse chemisorption was then carried out at room temperature by injecting 84 μL pulses of CO at regular intervals until the TCD signal reached saturation. The amount of chemisorbed CO was used to estimate the metal dispersion and calculate the surface copper area using a stoichiometry of CO:Cu = 1:1. The measurements were performed at 20°C after H_2_ reduction, assuming a 1:1 stoichiometry between CO and surface Cu atoms. Based on the amount of adsorbed CO, the copper dispersion (*D*, %) and Cu surface area (*S*_Cu_, m^2^/g) were calculated using the following equations:

(2) $$D=\frac{N_{Cu_{\text{surface}}}}{N_{Cu_{\text{total}}}}\times 100\%=\frac{n_{\mathrm{CO}}\times M_{w,\mathrm{Cu}}}{W\times m_{\mathrm{Cu}}}\times 100\%$$

(3) $$S_{\mathrm{Cu}}=\frac{N_{Cu_{\text{surface}}}\times A_{\mathrm{Cu}}}{W}=\frac{n_{\mathrm{CO}}\times N_{A}\times A_{\mathrm{Cu}}}{W}$$

where *n*_CO_ is moles of CO adsorbed (from pulse chemisorption), *M*_w,Cu_ is the molecular weight of copper (63.55 g/mol), *W* is the catalyst weight (g), wt% Cu is copper loading (60%), *N*_A_ is Avogadro's number (6.022 × 10^23^ mol^−1^), and *A*_Cu_ is the surface area per Cu atom (based on the Cu(111) surface structure, taken as 7.5 × 10^−20^ m^2^/atom).

**STEM** was employed to investigate the morphology and particle size distribution of the catalyst before and after the reaction. Samples were prepared by dispersing the catalyst in DI (deionized) water, followed by ultrasonication and deposition onto a carbon‐coated nickel TEM grid (carbon film only, 300 mesh, Ted Pella, Prod. #01843N). Imaging was performed on a Thermo Fisher Talos F200i S/TEM, operated at 200 kV, equipped with a Schottky field‐emission gun and a 16 MP Ceta CMOS camera. Bright‐field (BF) STEM imaging mode was utilized in this study to visualize the dispersion and size evolution of copper‐containing particles.

### 2.3 Catalytic Testing for Methanol Synthesis

**FIGURE 1:**
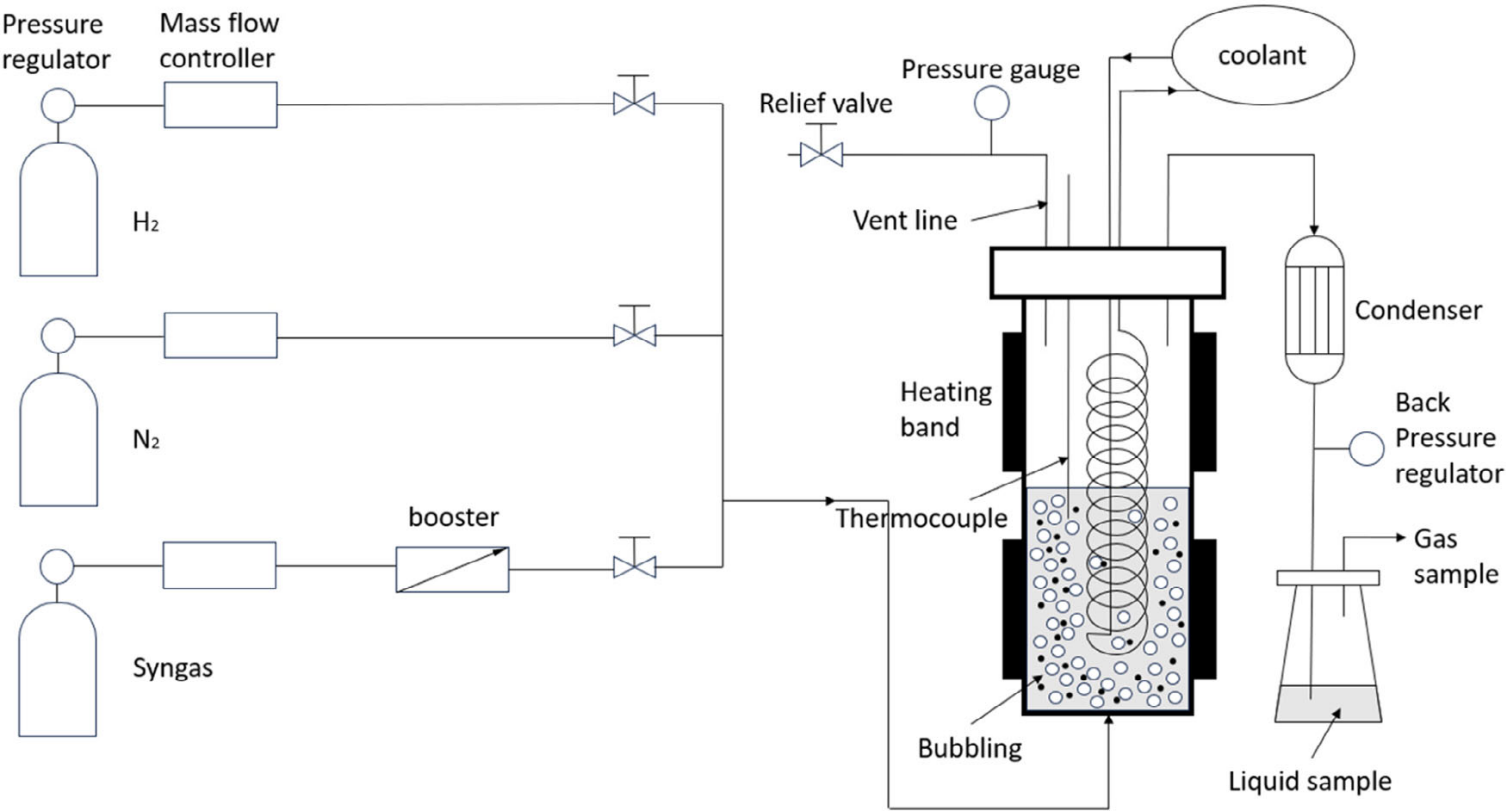
The scheme of the methanol synthesis reaction system.

Methanol synthesis reactions were performed in a custom‐designed stainless‐steel autoclave reactor (2 L internal volume), equipped with an external heating band, internal cooling coil, and dedicated gas inlet/outlet ports (Figure 1). The reactor was equipped with an internal thermocouple for direct temperature measurement. Pressure was controlled using a back‐pressure regulator, while an additional pressure gauge with a relief valve was installed at the reactor top to allow continuous monitoring and safe venting if required. At the bottom of the reactor, a porous stainless‐steel disc was installed. This disc served three functions: (i) it dispersed the incoming syngas feed into fine bubbles, thereby enhancing gas–liquid–solid contact; (ii) it mechanically supported the powdered catalyst during loading, while its fine pores ensured that no catalyst particles passed through into the gas inlet; and (iii) under normal operating conditions, the pressurized gas in the lines beneath the disc prevents downward leakage of liquid, so the disc effectively functions as a one‐way barrier. In each experiment, 30 g of the calcined CuO/ZnO/Al_2_O_3_ catalyst was loaded into the reactor. To remove residual air and moisture, the reactor was purged with UHP nitrogen (500 sccm) for 10 min at room temperature. The catalyst was then reduced in situ at ambient pressure by heating to 220°C under a flow of 10 vol% H_2_ balanced with N_2_ (800 sccm) for 20 h, in accordance with the optimal reduction conditions determined by H_2_‐TPR. After reduction, the reactor was cooled to room temperature under a continuous flow of nitrogen to prevent exposure to air.

Following reduction, the reactor was cooled to room temperature under a continuous N_2_ purge to stabilize the metallic Cu. During solvent addition, N_2_ flow was maintained, and the preprepared alcohol mixture (1000 mL) was quickly introduced through the top liquid inlet. Although complete exclusion of air could not be guaranteed, this procedure minimized oxygen exposure and ensured that the catalyst remained largely in its reduced state prior to reaction. Different isopropanol and 2‐butanol mixtures were used, with volume ratios of 100:0, 80:20, 50:50, 20:80, and 0:100 to investigate the effect of solvent composition on catalytic performance. The alcohols were used as received without further purification. Prior to initiating the reaction, the reactor was purged again with N_2_ to re‐establish an inert atmosphere after solvent addition and to minimize any possible catalyst oxidation during this step.

All gas‐handling components in the feed path (cylinders, regulators, mass‐flow controller, tubing, fittings) and reactor internals are stainless steel. Prior to catalyst reduction and solvent addition, the lines and reactor were purged with N_2_ to displace residual air and moisture, minimizing unintended surface oxidation or contamination during startup.

The reactor was then pressurized with syngas (H_2_:CO:CO_2_  = 65:30:5 vol%, which is a commonly used recipe in commercial methanol production) to an initial pressure of 5.0 MPa (725 psi) at ambient temperature. Upon reaching thermal equilibrium, the system was heated to 170°C while maintaining the reactor pressure at 5.0 MPa using a back‐pressure regulator. The gas feed was supplied at 1000 sccm, allowing for effective gas–liquid mixing through bubble agitation. No significant evaporation or loss of the liquid‐phase occurred throughout the reaction process. After 22 h of reaction, the reactor was cooled to room temperature and depressurized. The products and solvents were recovered from the reaction mixture via a distillation step. The liquid‐phase products were then analyzed using a Hewlett–Packard HP 5890 SERIES II gas chromatograph (GC) equipped with a TCD. An HP‐INNOWax capillary column (30 m × 0.32 mm ID × 0.25 μm film thickness) was employed. The carrier gas was He, and the flow rate was maintained at 22.2 mL/min.

The oven temperature of the GC was initially set at 40°C, held for 4 min, then ramped at 10°C/min to 120°C. The injection volume was 0.2 μL, and the sample was introduced in splitless mode. Quantitative analysis of methanol and reaction intermediates such as alkyl formates was performed using external calibration. Calibration curves were constructed by correlating peak areas with known molar quantities, allowing direct determination of the amount of each species in the reaction mixture. The effect of solvent composition on methanol yield, intermediate formation, and catalyst performance was systematically investigated. All measurements were repeated in duplicate to ensure reproducibility.

Based on the overall stoichiometry of methanol formation:

(4) $$\mathrm{CO}+2H_{2}\;\;\to \;{\mathrm{CH}}_{3}\mathrm{OH}$$

The methanol yield can be reflected using the following equation, with the number of moles of methanol produced and the total amount of carbon source (CO and CO_2_) supplied in the syngas feed:

(5) $$\text{Apparent methanol yield}=\frac{{\text{Methanol}}_{\text{produced}}}{{\text{Syngas}}_{\text{supplied}}\times \text{mol\%}(\mathrm{CO}+{\mathrm{CO}}_{2})}\times 100\%$$

## 3 Results and Discussion

### 3.1 Catalyst Reducibility

**FIGURE 2:**
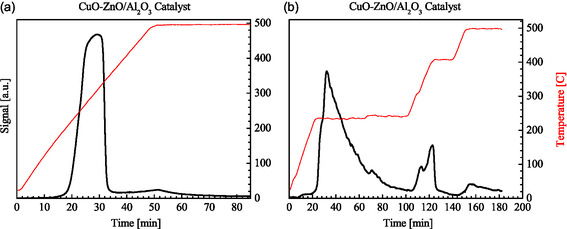
Hydrogen uptake (H_2_‐TPR profiles) as a function of time obtained from the CuO/ZnO/Al_2_O_3_ catalyst during (a) heating at 10°C per min up to 500°C and (b) a programmed ramp with a heating rate of 10°C per min with a 80 min hold at 230°C and a 20 min hold at 400°C.

The reducibility of the copper species in the commercial CuO/ZnO/Al_2_O_3_ catalyst was studied using H_2_‐TPR. These measurements can yield information on oxidation states, active metal dispersion, and metal–support interactions. Since the active sites in methanol synthesis are metallic Cu sites, these measurements are also important for determining the reduction temperature required for catalyst activation. The hydrogen uptake as a function of time during a constant temperature ramp from room temperature to 500°C is shown in Figure 2a. One major peak centered around 300°C is observed, accompanied by a smaller, less pronounced peak as the temperature reaches 500°C. The main peak corresponds to the reduction of accessible CuO species to metallic Cu, while the second feature is likely due to copper species interacting closely with the alumina support, potentially CuAl_2_O_4_ species, which are more difficult to reduce.

It is essential to note that, at a constant heating rate, the temperature at which maximum hydrogen uptake is observed is typically higher than the temperature required for reduction under isothermal conditions. This is due to the inherent lag between the furnace temperature increase, the catalyst temperature and the TCD signal response. When selecting the reduction temperature, this must be considered, particularly since sintering of the metallic copper can occur at higher temperatures [34]. Therefore, the lowest temperature that will prevent the reduction of CuO on the surface of the catalyst should be chosen to avoid sintering of the Cu metal that forms during reduction. According to the TPR data, reduction is initiated around 230°C (Figure 2a). Therefore, this temperature was selected as the initial reduction temperature, and the hydrogen uptake was monitored during heating at a rate of 10°C/min up to 230°C (Figure 2b). After holding the temperature at 230°C for 80 min, the temperature increased to 400°C (at 10°C/min) and was held at this temperature for 20 min.

From Figure 2b, it is evident that a temperature of 230°C is sufficient to reduce most of the CuO on the surface of the catalyst to metallic Cu. It suggests that a large portion of CuO is present in a relatively accessible and reducible form. The broader peak during reduction at 230°C compared to the hydrogen uptake curve during heating at a constant rate to 500°C reveals that the reduction is slower during isothermal conditions at 230°C. As the temperature increased from 230°C to 400°C, additional hydrogen was consumed. Therefore, a temperature of 230°C is not sufficient to reduce all the CuO on the Al_2_O_3_‐supported catalysts. Under these conditions, a distinct feature is also observed closer to 400°C. This could be due to strong interactions between the CuO and the Al_2_O_3_ support, potentially due to the formation of CuAl_2_O_4_‐type spinel phases (or possibly Cu ions incorporated into zinc aluminate, Cu_*x*_Zn_1‐*x*_Al_2_O_4_). Such species are known to exhibit higher thermal stability, hence requiring elevated temperatures for reduction [35, 36].

While strongly bound species are often described as catalytically inactive due to their oxidized form, they can become active once reduced to Cu [37]. However, reduction at elevated temperatures may lead to undesirable particle growth and sintering, which decreases the overall copper dispersion and, therefore, the activity of the catalyst. To balance these considerations, a reduction temperature of 220°C was selected for the pretreatment of all the catalysts. This condition ensures a high extent of reduction of the accessible CuO phase while minimizing the risk of sintering associated with higher‐temperature treatment.

### 3.2 Liquid‐Phase Methanol Synthesis

**FIGURE 3:**
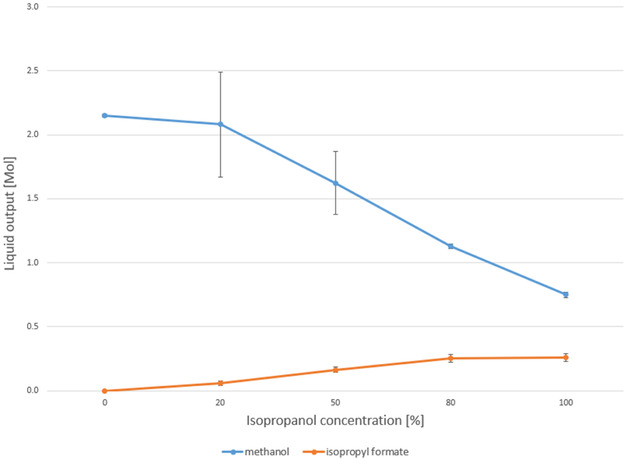
Total amount of liquid obtained after 22 h of methanol synthesis.

To investigate the effect of alcohol solvent on methanol synthesis, a series of reactions was conducted using mixtures of 2‐butanol and isopropanol. Alcohol solvents with isopropanol volume fractions varying between 0%, 20%, 50%, 80%, and 100% were investigated, with the remaining fraction being 2‐butanol. The total liquid volume (1.0 L), operating conditions (170°C, 5.0 MPa), and reaction time (22 h) were kept constant in all experiments. Each experiment consumed about 1.2 m^3^ (reported at standard temperature and pressure (STP)) of syngas. The molar quantities of methanol and reaction intermediates in the condensed liquid phase were quantified by GC‐TCD and are plotted in Figure 3.

The maximum amount of methanol (2.15 mol) was produced when 2‐butanol was used as the solvent (0% isopropanol). Under these conditions, no intermediate or product other than methanol was detected. As the isopropyl alcohol concentration increased, the methanol yield decreased. The nearly linear trend with alcohol composition indicates that there are no synergistic (or antagonistic) interactions between them under these conditions. Another product, an isopropyl formate intermediate, was also observed when isopropanol was present, and its concentration increased with the isopropanol content. When 100% isopropanol was used as the solvent, the methanol produced was only 0.75 mol, and the intermediate product reached its maximum concentration (0.26 mol).

**FIGURE 4:**
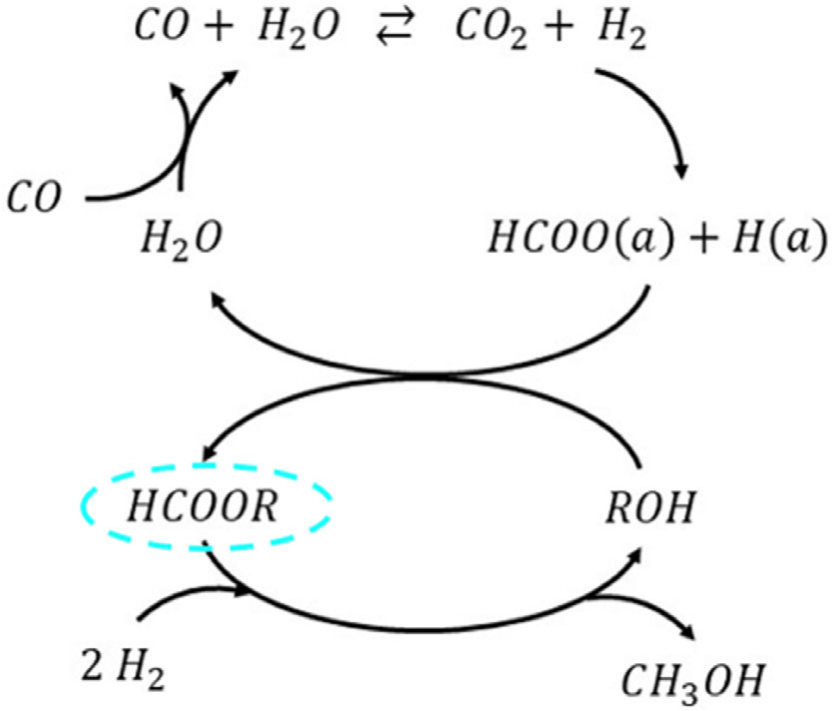
Illustration of methanol synthesis pathway via formate ester route (with the ester intermediate circled).

The formation of isopropyl formate as the only detectable intermediate suggests that isopropanol participates in the reaction and methanol formation proceeds via a formate ester route (Figure 4). In contrast, no 2‐butyl formate was observed in any reaction. This could indicate that 2‐butanol either follows a different, possibly more direct pathway to methanol or that the 2‐butyl ester intermediate is more reactive and is rapidly consumed under the applied conditions. It is difficult to imagine that the extra methylene group of 2‐butanol (compared to 2‐propanol) would result in a different reaction pathway. It is therefore more likely that the observed trends are due to reactivity differences of the intermediates.

The solvent‐dependent appearance of isopropyl formate only when isopropanol is present, together with the monotonic decrease in methanol yield as the isopropanol fraction increases, is consistent with the commonly accepted alcohol‐assisted formate pathway on Cu/ZnO under low‐temperature liquid‐phase conditions. Prior in situ DRIFTS and liquid‐phase studies have shown that CO/CO_2_ hydrogenation proceeds via surface formate species on Cu/ZnO, which can be trapped by the alcohol as alkyl formates and subsequently hydrogenolyzed to methanol [16, 22, 23, 24, 26, 38]. In our system, the stabilization of isopropyl formate plausibly retards hydrogenolysis and partially blocks Cu sites, lowering net methanol productivity, whereas 2‐butanol does not exhibit a detectable 2‐butyl formate, consistent with a more labile intermediate and higher methanol production.

It is important to note that even if all of the isopropyl formate was reduced to methanol, the methanol yield would still be lower when isopropyl alcohol is used as the solvent. This suggests that the slower reduction of isopropyl formate to methanol not only allows the intermediate product to desorb from the catalyst surface but also likely blocks active sites, thereby decreasing the methanol yield.

The methanol‐based conversion ranged from 11.5% (in the pure 2‐butanol system) to 4.0% (in the pure isopropanol case). While these numbers may appear modest, it is important to note that this is a one‐pass conversion, and no mechanical stirring was employed in the reactor. Gas–liquid mixing relied solely on the bubbling of syngas through the alcohol solvent, meaning that a significant fraction of the syngas served merely as purging gas, providing agitation, bypassing the liquid–solid interface without participating in the reaction. As such, the actual local conversion at the gas–liquid–solid boundary may be substantially higher.

### 3.3 Catalyst Structure

**FIGURE 5:**
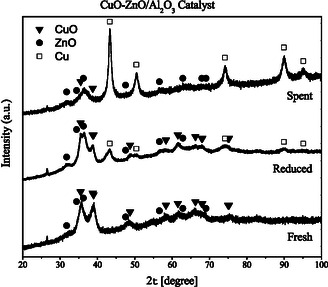
XRD patterns of the CuO/ZnO/Al_2_O_3_ catalyst as received (fresh), after reduction at 220°C (reduced), and after exposure to reaction conditions (spent). Symbols indicate reference positions of the reflections from the ICDD Powder Diffraction File (PDF) for the phases observed: CuO (▪, tenorite, monoclinic, PDF#45‐0937) with representative 2*θ* (Cu K*α*) peaks at ~32.5°, ~35.5°, ~38.7°, ~48.7°; ZnO (▴, wurtzite, hexagonal, PDF#36‐1451) with representative peaks at ~31.8° (100), ~34.4° (002), ~36.2° (101), ~47.5° (102); and Cu (•, fcc, PDF#04‐0836) with representative peaks at ~43.3° (111), ~50.4° (200), ~74.1° (220). Note that the 36°–37° region can exhibit contributions from multiple phases: ZnO (~36.2°), residual CuO (~35.5°), and possible spinel‐type aluminates (CuAl_2_O_4_/ZnAl_2_O_4_) near ~36.8°. Quantitative crystallite sizes discussed in the text were obtained by Scherrer analysis using the CuO and Cu reflections indicated.

XRD was employed to analyze the phase composition of the commercial CuO/ZnO/Al_2_O_3_ catalyst before and after reduction, as well as after reaction experiments (Figure 5). The diffraction pattern obtained from the fresh catalyst reveals rather broad and weak diffraction peaks and a rather noisy background signal, indicating poor crystallite alignment and a low signal‐to‐noise ratio. The dominant crystalline phase observed is monoclinic CuO, but small peaks corresponding to hexagonal ZnO are also visible. As expected, the *γ*‐Al_2_O_3_ phase of the support cannot be detected, as high surface area alumina is typically poorly crystalline (or amorphous) in nature. Furthermore, only the most intense peaks of the CuO and ZnO phases are observed due to the low crystallinity of the catalyst. According to the Scherrer equation, the crystallite sizes of CuO in the fresh catalyst are ≈9.9 and 10.3 nm, based on the (002) and (111) reflections, respectively. This indicates a polycrystalline sample, in which CuO particles consist of fine CuO crystallites.

The CuO/ZnO/Al_2_O_3_ catalyst was reduced in the reactor at 220°C (as described in the Experimental section) and then cooled down in an inert atmosphere before being transferred for XRD analysis. While some reoxidation of the catalyst likely occurs during sample transfer, it is expected that this is limited to the catalyst's surface, which may not be detectable by XRD. The reductive treatment introduces peaks due to metallic Cu and reduces the contribution from CuO. However, a significant amount of CuO remains after reduction under these conditions. The peaks due to the hexagonal ZnO also appear more intense after this treatment, suggesting some growth of crystallites or particles. The close proximity of the major peaks due to CuO (2*θ* = 35.495°) [39, 40] and ZnO (2*θ* = 36.252°) [40, 41] makes it difficult to estimate changes in crystallite size before and after the reduction treatment. However, using the (002) and (111) reflections, the crystallite sizes for CuO in the reduced catalyst are estimated to be 16.3 and 13.3 nm, respectively. These values were obtained directly from the broadened peaks without deconvolution of the overlapping ZnO and potential spinel contributions. Therefore, they should be regarded as approximate lower‐limit estimates of the actual crystallite size, and the true extent of crystallite growth may be even greater. Therefore, despite the reduction of CuO to Cu metal, there is crystallite growth even at 220°C. The crystallite size of the metallic Cu in the reduced catalyst is estimated to be 10.2 nm, as determined by the Scherrer equation and using the Cu(111) peak at 43.3°. In addition, a new peak appears at 2*θ* = 36.5°, and while this could be due to an increased contribution from ZnO (2*θ* = 36.252°), since the contribution from CuO is reduced, the presence of spinel‐type aluminate phases cannot be excluded. Both copper and zinc aluminates have the major reflections around 2*θ* = 36.8° (CuAl_2_O_4_ (2*θ* = 36.867°) [42, 43] and ZnAl_2_O_4_ 2*θ* = (36.836°) [40]. The second largest reflection of CuAl_2_O_4_ (2*θ*= 31.294°) [42, 43] and ZnAl_2_O_4_ 2*θ* = (31.237°) [40] also overlaps with the ZnO peak at 2*θ* = 31.769°. Overall, the sample is more crystalline after the reductive treatment, since the signal‐to‐noise ratio is higher after this treatment, but due to the broad and overlapping peaks, it is still difficult to unambiguously confirm the presence of aluminates.

After exposure to reaction conditions, it is evident that most of the CuO originally present in the catalyst has been reduced to metallic copper (Figure 5). In fact, it is difficult to detect CuO in the XRD pattern obtained from this catalyst. Most of the peaks remaining in the XRD pattern obtained from this catalyst are due to metallic Cu and ZnO (Figure 5). This indicates that, during the syngas‐to‐methanol process, any CuO species present in the fresh catalyst—whether incompletely reduced or reoxidized prior to the reaction—were reduced to metallic Cu by the H_2_ (or CO) in the syngas stream. Therefore, even though the reduction temperature selected was not sufficient to reduce all CuO in the catalyst to Cu metal, it is evident that the remaining CuO can be reduced during reaction to generate active metallic Cu. According to the Scherrer equation, the crystallite size of metallic copper after exposure to reaction conditions is ≈11.8 nm. This represents only minor crystallite growth under the LT‐LPMS conditions. Only the most intense ZnO peaks are visible in the XRD pattern after reaction. The peak centered around 2*θ* = 36.5° appears to have contributions from two phases, likely the ZnO at 2*θ* = 36.2° and CuAl_2_O_4_ or ZnAl_2_O_3_ at 2*θ *= 36.8°.

The XRD data indicate that, although the reduction pretreatment does not completely remove all the CuO from the catalyst, the remaining CuO is fully reduced to metallic Cu during the reaction. Minor crystallite growth is also observed; however, it is expected that this is significantly less than the sintering that would occur during high‐temperature gas‐phase synthesis.

**CO pulse chemisorption** was employed to evaluate the surface metallic copper sites of the fresh and spent CuO/ZnO/Al_2_O_3_ catalysts. Based on the volume of CO chemisorption, the calculated Cu surface area for the fresh catalyst was 1.02 m^2^/g, which corresponds to a copper dispersion of 0.27%. The Cu surface area after reaction was determined to be 0.37 m^2^/g, which corresponds to a 0.10% dispersion. These absolute values are notably low but are expected due to the high copper content of the catalyst. At Cu loadings exceeding 60% by weight, the initial surface‐to‐volume ratio of Cu is low on the fresh catalyst. Additionally, there is limited support available to prevent metal particle growth and agglomeration during the reaction, which further reduces the fraction of exposed surface atoms. Furthermore, CO binds relatively weakly to Cu surfaces under experimental conditions, which can lead to an underestimation of the number of surface Cu atoms. While N_2_O chemisorption is often used to determine copper surface areas, CO adsorption was used in this study primarily to detect changes in copper sites before and after the reaction. Loss in copper surface area during reaction could be due to sintering of metallic Cu particles, formation of inactive oxides, such as CuAl_2_O_4_, or dissolution of copper species in the liquid phase. Furthermore, the presence of surface contaminants, for example, carbonaceous deposits that occur during reaction, can block Cu sites and reduce the amount of CO adsorbed even if the Cu content remains the same. The significant drop in accessible Cu surface sites likely contributes to the observed deactivation during reaction.

**FIGURE 6:**
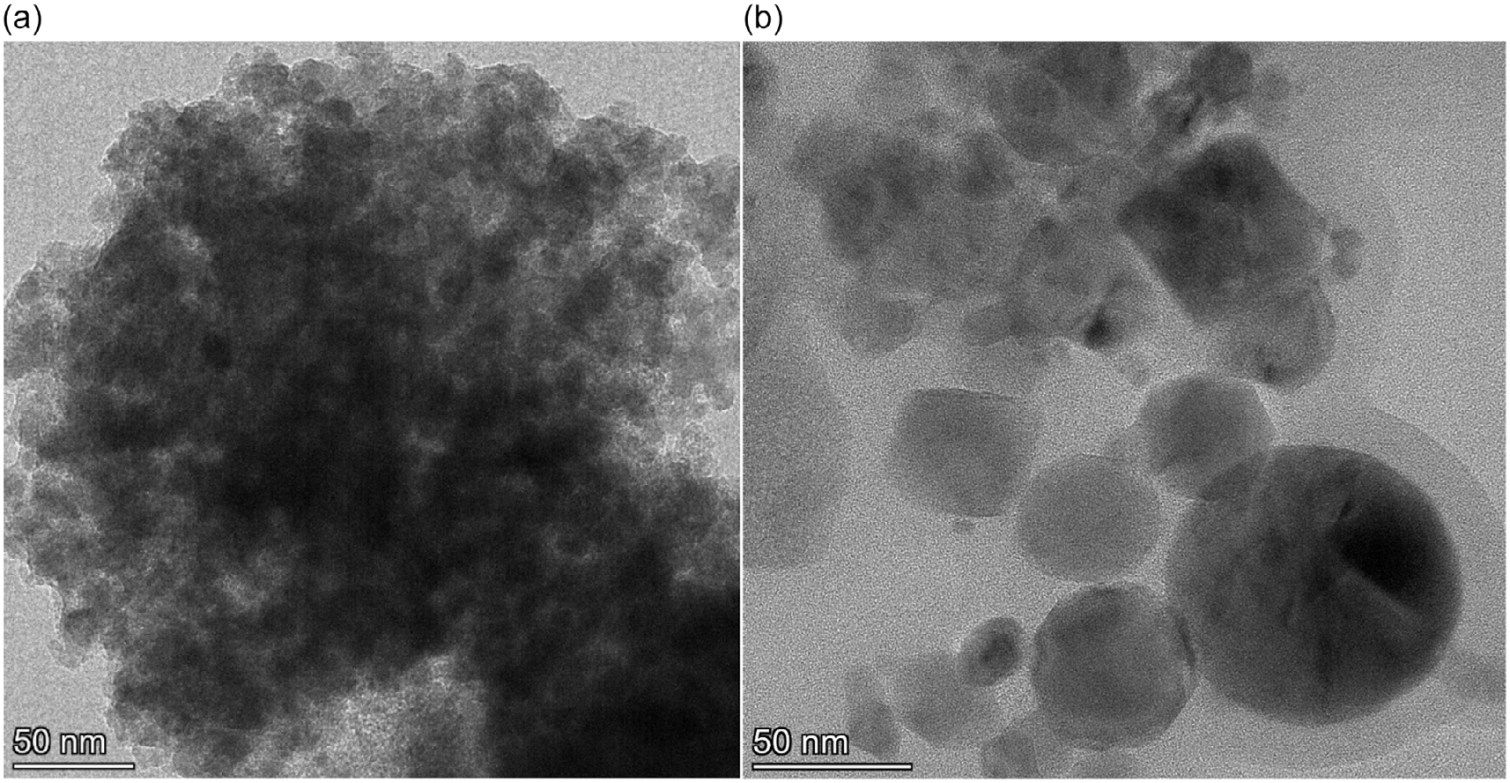
S/TEM images of the CuO/ZnO/Al_2_O_3_ catalyst, (a) fresh and (b) spent. Scale bars: (a) 50 nm, (b) 50 nm. Particle sizes were estimated by manual measurement of clearly resolved particles across multiple fields of view; fresh: 13 ± 4 nm; spent: 30 ± 20 nm, with occasional particles near ~100 nm. These images support qualitative coarsening; quantitative crystallite sizes are provided by XRD–Scherrer analysis in the text.

**S/TEM** was employed to observe the particle morphology of the fresh and spent CuO/ZnO/Al_2_O_3_ catalysts. The S/TEM image obtained from the fresh catalyst reveals a sample consisting of fairly small and agglomerated particles with an average diameter of around 13+/− 4 nm (Figure 6). The distribution of CuO, ZnO, and Al_2_O_3_ in the catalyst is expected to be relatively uniform.

After the reaction, significant restructuring has occurred. The particles are significantly larger and have a wider size distribution. The average particle size is around 30 nm with a standard deviation of 20 nm, and a few particles close to 100 nm can be observed. This increase in size indicates that particle growth and aggregation occur even during low‐temperature methanol synthesis.

Overall, the particle growth observed is more extensive than expected from the CO chemisorption experiments and the XRD analysis.

## 4 Conclusion

This work investigates a LT‐LPMS system using commercial CuO/ZnO/Al_2_O_3_ catalysts and mixed alcohol solvents under 170°C and 5.0 MPa. The study demonstrates that the alcohol solvent affects methanol yield and product selectivity. Among tested alcohols, 2‐butanol provided the highest methanol productivity, likely due to the formation of a more reactive intermediate. In contrast, the presence of isopropanol led to substantial accumulation of isopropyl formate, indicating that its conversion to methanol was kinetically hindered.

Postreaction characterization revealed moderate sintering and copper particle growth, yet without dramatic deactivation, suggesting that the system is structurally robust under the applied conditions. The use of 30 g of catalyst—significantly more than is typically used in lab‐scale evaluations—enables insights into performance under semirealistic, scaled‐up settings. Furthermore, agitation driven purely by syngas bubbling (without mechanical stirring) highlights the mass transfer challenges in such systems and provides context for the moderate methanol conversions observed. XPS/AES could provide valuable insight into the near‐surface Cu^0^/Cu^+^ balance. However, ex situ XPS/AES on liquid‐phase catalysts is susceptible to air‐exposure artifacts. And in situ XPS under liquid‐phase conditions is hard to achieve, we opted not to conduct XPS analyses.

Altogether, the results provide a framework for understanding how different alcohol solvents directly influence the reaction pathway: 2‐butanol promotes the formation of productive intermediates that enhance methanol yield, while isopropanol stabilizes less reactive esters that hinder conversion. These findings highlight the critical role of solvent choice in tuning intermediate stability and product selectivity, while also demonstrating that the CuO/ZnO/Al_2_O_3_ catalyst is an effective catalyst under these liquid‐phase conditions. Such insights can guide the rational design of liquid‐phase synthesis routes and the selection of solvent–catalyst combinations for efficient low‐temperature C1 chemistry.

Beyond reaction chemistry, the broader implications of this work align with RRG, an advanced recycling framework that converts heterogeneous waste streams into syngas upstream and channels methanol downstream into polymers, packaging, and other circular products. Methanol serves as a versatile platform chemical for circular manufacturing, enabling integration with established petrochemical routes such as MTO and oxygenate synthesis.

For energy applications, only the biogenic fraction of waste‐derived methanol qualifies for renewable fuel crediting under current regulatory frameworks, whereas fossil‐derived methanol should be retained in closed‐loop products to avoid re‐emission. When both fossil and biogenic carbon streams are committed to durable goods and RRG facilities operate on carbon‐free energy, these systems can function as operational carbon sinks, embedding carbon in long‐lived products while displacing virgin petrochemical inputs.

Altogether, this work provides mechanistic insight into solvent‐controlled pathways and situates LT‐LPMS as a deployable unit operation within RRG, advancing circular carbon strategies under industrially relevant conditions.

## Funding

This study was supported by Consortium for Waste Circularity (CWC‐02).

## Conflicts of Interest

The authors declare no conflicts of interest.
